# Phylodynamic and phylogeographic analysis of the complete genome of the West Nile virus lineage 2 (WNV-2) in the Mediterranean basin

**DOI:** 10.1186/s12862-021-01902-w

**Published:** 2021-09-27

**Authors:** Haythem Srihi, Noureddine Chatti, Manel Ben Mhadheb, Jawhar Gharbi, Nabil Abid

**Affiliations:** 1 Research Unit UR17ES30 “Genomics, Biotechnology and Antiviral Strategies”, Higher Institute of Biotechnology of Monastir, University of Monastir, Tahar Hadded Avenue, PB 74, 5000 Monastir, Tunisia; 2 Laboratory of Transmissible Diseases and Biological Active Substances LR99ES27, Faculty of Pharmacy, University of Monastir, Ibn Sina Street, 5000 Monastir, Tunisia; 3 High Institute of Biotechnology of Sidi Thabet, Department of Biotechnology, University of Manouba, BiotechPôlet Sidi Thabet, PB 66, 2020 Ariana-Tunis, Tunisia; 4 Department of Biological Sciences, College of Science, King Faisal University, PB 400, Post Code 31982 Al-Ahsa, Saudi Arabia

**Keywords:** West Nile virus lineage 2, Spatio temporal phylodynamics, Bayesian phylogeography, Migratory birds

## Abstract

**Background:**

The West Nile virus is a highly contagious agent for a wide range of hosts. Its spread in the Mediterranean region raises several questions about its origin and the risk factors underlying the virus’s dispersal.

**Materials and methods:**

The present study aims to reconstruct the temporal and spatial phylodynamics of West Nile virus lineage 2 in the Mediterranean region using 75 complete genome sequences from different host species retrieved from international databases.

**Results:**

This data set suggests that current strains of WNV-2 began spreading in South Africa or nearby regions in the early twentieth century, and it migrated northwards via at least one route crossing the Mediterranean to reach Hungary in the early 2000s, before spreading throughout Europe. Another introduction event, according to the data set collected and analyses performed, is inferred to have occurred in around 1978. Migratory birds constitute, among others, additional risk factors that enhance the geographical transmission of the infection.

**Conclusion:**

Our data underline the importance of the spatial–temporal tracking of migratory birds and phylodynamic reconstruction in setting up an efficient surveillance system for emerging and reemerging zoonoses in the Mediterranean region.

**Supplementary Information:**

The online version contains supplementary material available at 10.1186/s12862-021-01902-w.

## Background

The West Nile virus (WNV) was initially isolated from a woman presenting a febrile syndrome in Uganda in 1937 [68]. The virus was not correlated with severe human disease until the 1990s, when outbreaks in the Americas, Europe, the Middle East, and other areas were associated with higher rates of West Nile neuroinvasive disease. Furthermore, the unexpected emergence in the Americas and Europe with different prevalence rates has changed the attention paid to this virus and stressed the role of ecological, viral, and host factors in the differential emergence of WNV in the world. In Africa, the most important WNV outbreak occurred in 1974 in South Africa, where WNV is considered to be endemic and maintained through an enzootic cycle between different avian species and *Culex univittatus* mosquitoes [34].

WNV is primarily transmitted by mosquitoes. Members of the genus *Culex* are the main vectors worldwide, although other mosquito genera can also be infected [45]. WNV has been detected in many regions, including Africa, Europe, Asia, the Middle East, Australia, America, and the Caribbean [55, 61]. Currently, it has become the most widely distributed of the encephalitic flaviviruses [13].

Two major lineages, lineages 1 and 2 (WNV-1 and WNV-2), were identified in Africa [9], following the first isolation in Uganda, they are considered endemic in Europe (https://www.ecdc.europa.eu/en/west-nile-fever/facts/factsheet-about-west-nile-fever).

WNV-1 occurs mainly in central and northern Africa, Europe, Australia, and the Americas [20], whereas WNV-2 is endemic in southern Africa and Madagascar; yet emerged in central Europe in 2004, where it was isolated from the brain of a goshawk (*Accipiter gentilis*) in Hungary [5]. A human case of WNV-2 infection was retrospectively confirmed to have occurred in Russia in the same year [56]. Nevertheless, the first evidence of WNV-2 in Cyprus stems from a serological and ectoparasite survey of migratory birds in the Eastern Mediterranean between 1966 and 1971 [77], raising the question of its real introduction date into Europe. Subsequent outbreaks have occurred in a number of other European countries from 2004 to 2018, including Austria, Greece, Romania, Serbia, Italy, Spain, and Germany [10, 49, 54, 67, 82].

Less common lineages (lineage 3, known as Rabensburg virus; lineage 4 in Russia; lineage 5 in India; lineage 6 in Spain), likely evolved from separate introductions into the Northern Hemisphere [61]; further lineages are being discovered in Africa [20]. However, lineages 1 and 2 are still the most important from a public health standpoint, causing epidemics in North America and Europe [29, 80]; they are under-reported in Africa.

The phylodynamic approach may constitute a reliable method to describe the correlations between the epidemiology and evolutionary processes of viruses, thus allowing the reconstruction of the history of an infectious agent on the basis of the phylogeny of sampled sequences [76].

The aim of the present study was the dynamic analysis of WNV-2 using the available 75 full genome sequences collected from humans, mosquitos, and other animal species. In particular, we focused on the viral strains circulating in Africa and Europe. Bayesian methods were performed for phylodynamic and phylogeographic approaches. Additionally, we highlight the importance of the surveillance of migratory birds crossing Africa and Europe for WNV-2 dispersal, as they constitute an additional risk factor in the emergence and spread of the virus. While this study is not meant to be an exhaustive examination of the sequence data available for WNV-2, our analysis demonstrates the value of phylodynamics in studies on the virus and a plausible path of their spread.

## Materials and methods

### Data sources and collection

The available complete genome sequences of WNV-2 were retrieved from GenBank (http://www.ncbi.nlm.nih.gov/, until 17/05/2019). We included human and animal strains collected from different geographical regions at different sampling dates. Viral sequences were selected according to the following inclusion criteria: (1) sequences had already been published in peer-reviewed journals; (2) there was no uncertainty about the assigned type of each sequence and they were classified as non-recombinant (see Sect. “Recombination and substitution model analysis”); (3) when applicable, only one sequence per infected family was randomly selected; and (4) the city/state of origin and sampling date were known and clearly established in the original publication. We removed sequences with a hypermutation and an internal stop codon or an ambiguous nucleotide. Out of 247 full genome sequences prior to the filtration step, 75 sequences were retained. The sequences were obtained from mosquitoes (n = 17), birds (n = 15), horses (n = 5) and humans (n = 38). The sampling period spanned 60 years (from 1958 to 2018) and included South Africa (n = 4), Italy (n = 14), Ukraine (n = 1), Serbia (n = 7), Hungary (n = 3), Greece (n = 9), Austria (n = 24), Senegal (n = 1), Czech Republic (n = 4), Bulgaria (n = 1), Zambia (n = 1), Belgium (n = 1), Slovakia (n = 2), and Germany (n = 3). The viral sequences were selected and annotated using the Sequence Name Annotation-based Designer (SNAD) [66]. Sequence annotation includes accession number, country, and date of collection. The annotations were further checked manually for errors and the accession numbers were replaced by their appropriate annotations using Javascript (Additional file 1: Table S1). Additionally, we retrieved the available scientific data on the geographic distribution of several migratory birds. The spatial data was obtained from the International Union for Conservation of Nature (IUCN, January 2019) and then used to reconstruct a geographic map using Google Earth (www.google.com/earth/). Datawrapper (https://www.datawrapper.de/) and EMMA (https://emma.ecdc.europa.eu) were used to visualize the data, which are simple web-based GIS (Geographic Information System) tools that support the creation of maps that can be used to identify patterns in communicable disease surveillance data or during outbreak investigations. The available data were collected from 2008 until October 2018 (Additional file 1: Figure S1).

### Recombination and substitution model analysis

The detection of recombination before carrying out phylogenetic analysis constitutes a crucial step for a given set of aligned sequences. Recombination Detection Program 4 (RDP4) [44] was used to inspect the used sequences for possible recombination, with a window size of 100 nucleotides (and default parameters),it includes seven published recombination detection methods in a single suite of tools.Some methods are phylogeny-based (BootScan, RDP, and Siscan) while others are substitution-based methods (GenConv, Maxchi, Chimera and 3SEQ). Statistical evidence of recombination was indicated by *p*-values < 0.05, after Bonferroni correction for multiple comparisons. Putative recombination events were considered significant if supported by at least five of the seven algorithms.

### Likelihood mapping analysis

The sequences were aligned using ClustalX software [38] and edited manually for optimization. Though the accuracy of an alignment cannot be measured directly, it can be indirectly assessed via the phylogenetic content of the data. The phylogenetic content in our data set was evaluated by likelihood-mapping that calculates maximum-likelihood (ML) trees for all possible quartets of sequences and counts the frequency of trees according to their quality. We computed quartet weights using default parameters in the Tree-Puzzle program [69]. A total of 10,000 random quartets (groups of four randomly chosen sequences) were evaluated and, for each quartet, the three possible unrooted trees were reconstructed using the ML approach under the selected substitution model. Using the Hasegawa–Kishino–Yano model of substitution [27], the posterior probabilities of each tree were then plotted on a triangular surface; fully resolved trees fall into the corners and the unresolved quartets in the center of the triangle. When more than 30% of the dots fall into the center, indicating a star-like signal, the data are considered unreliable for phylogenetic inference.

### Assessing the temporal structure of WNV-2

We conducted a range of analyses to assess the extent of temporal structure in the data and to estimate the rate and time-scale of WNV-2 evolution. Data sets must possess temporal structure for tip-dated analyses to be informative [60]. Therefore, to initially verify the temporal structure in the data, we conducted regressions of root-to-tip genetic distance as a function of the sampling time (year) using TempEst v0.1 [57] using a ‘non-clock’ ML phylogenetic tree (see section below). To further assess the extent of temporal structure, we employed a Bayesian date-randomization test, in which the nucleotide substitution rate is estimated using the correct sampling dates (Standard), and the analysis was repeated 10 times on a data set in which the sampling dates had been randomized among the sequences [58].

### Phylogenetic analysis

The substitution model for the given data was evaluated using phylogenetic analysis using the PAUP * ver 4 software [71]; the best model that fits the data best was chosen using the Bayesian information criterion (BIC) score [64].The ML phylogenetic tree was inferred with the IQTree ver 1.6.12 software [51], using the general time reversible (GTR) + G + I nucleotide substitution model and a combination of nearest neighbor interchange (NNI) [50, 62] and subtree pruning and regrafting (SPR) [28] rearrangement strategies. ML tree reliability was evaluated using four methods: approximate Bayes test [3], ultrafast bootstrap (UFBoot) [30], Shimodaira–Hasegawa-like approximate likelihood ratio test (SH-like aLERT) [24], and local bootstrap probability (LBP) [1].

Bayesian genealogies were also inferred with the BEAST ver 1.8.2 software package [16, 17] launched on an online cluster (bioinfo-nas.ird.fr) using the GTR + G + I substitution model, an uncorrelated lognormal relaxed clock, and the Bayesian skyline plot (see section below). The Bayesian Markov Chain Monte Carlo (MCMC) was run for 100 million generations with a sampling step every 20,000 generations. The results were evaluated and visualized using Tracer ver. 1.6 (http://tree.bio.ed.ac.uk/software/tracer/).

The MCMC is considered resolved with an effective sample size (ESS) value of greater than 200, indicating sufficient mixing of the Markov chain. The maximum clade credibility (MCC) tree was then selected from the posterior tree distribution using TreeAnnotator ver 4.1.8, available within the BEAST software package. The generated tree was visualized and annotated with FigTree ver 1.4.2 (http://tree.bio.ed.ac.uk/software/figtree).

### Clock assumption and coalescent prior models’ selection

Different clock models (strict molecular clock and uncorrelated lognormal relaxed clock) and different coalescent priors (constant population size, exponential growth, Bayesian skyline plot, and Bayesian Skygrid model) were evaluated by their Bayes factors (BF) [70], allowing us to choose the best-fit population dynamics model. The BF value is the ratio of the marginal likelihoods (marginal with respect to the prior) of the two models being compared [70]. We calculated approximate marginal likelihood estimators (MLE) to compare path sampling (PS) [39] and stepping-stones sampling (SS) methods [4, 78]. The strength of the evidence against H_0_ (null hypothesis) was evaluated as follows: 2 lnBF < 2 no evidence; 2–6 weak evidence; 6–10 strong evidence, and > 10 very strong evidence [35]. A negative 2 lnBF indicates evidence in favour of H_0_. Only 2 lnBF values of ≥ 6 were considered significant.

### Phylogeographic analysis

The phylogeographic inferences of WNV complete genomes were analyzed and visualized using Spatial Phylogenetics Reconstruction of Evolutionary Dynamics using Data-Driven Documents (D3) (SPREAD3) ver. 0.9.7.1 (https://rega.kuleuven.be/cev/ecv/software/SpreaD3) [7]. The generated keyhole markup language (KML) file, generated by SPREAD3, was used to visualize the geographic migration of the virus over time using Google Earth (www.google.com/earth/). The resulting log files were used to calculate BF values for significant diffusion rates between discrete locations. A Bayesian stochastic search variable selection (BSSVS) approach was used to find a minimal (parsimonious) set of rates explaining the diffusion in the phylogeny, which allows the migration rates in the continuous-time Markov chain (CTMC) to be zero with some prior probability [35]. A BF > 3 was considered suggestive of a significant migration pattern between country pairs.

## Results

### Likelihood mapping and Temporal signal analysis

**Fig. 1:**
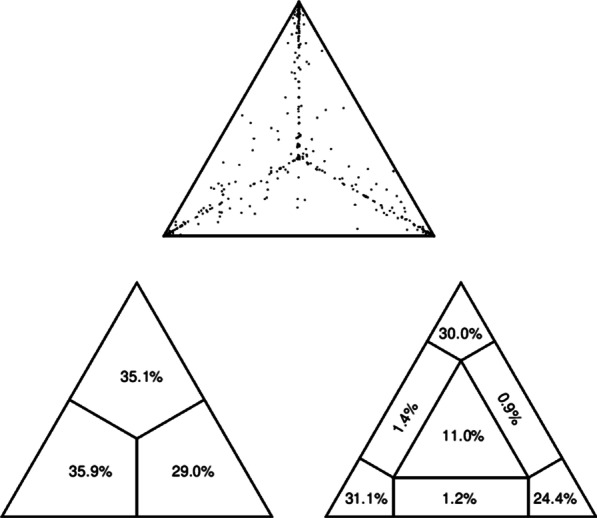
Likelihood mapping of the complete genome WNV-2 worldwide strains used in our study. Each dot represents the likelihood of three possible tree topologies for a group of four sequences (quartets) chosen randomly from the dataset. The dots localized close to the triangle vertices represent the tree-like phylogenetic signal. Those in the centre and on the laterals represent the star-like and network-like signals, respectively. The numbers inside the triangles represent the percentage of dots plotted in the centre, laterals, and the vertices

**Fig. 2:**
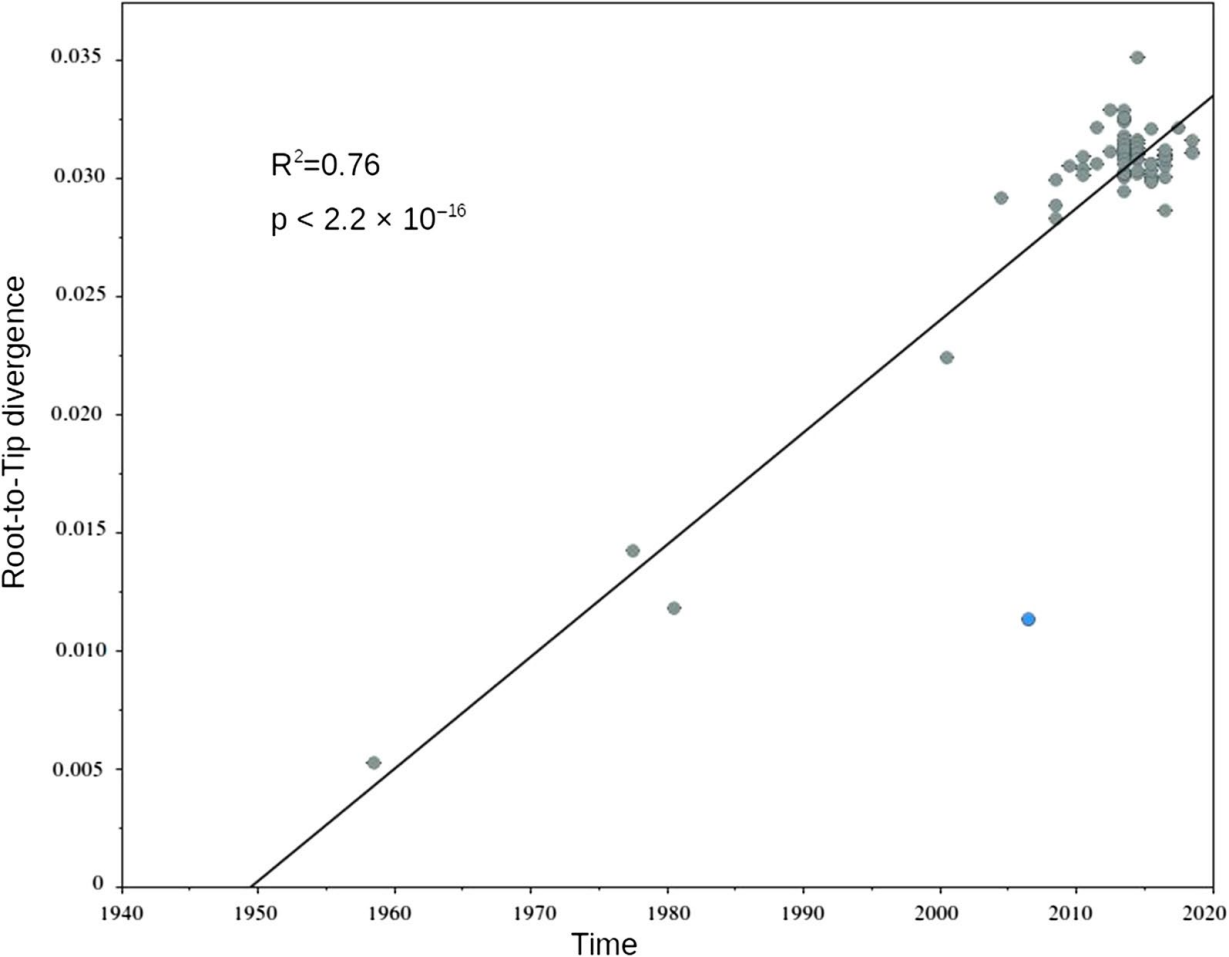
Results of the regression of root-to-tip distance against sampling date. A significant positive correlation is consistent with the presence of temporal signal. *p-value* were obtained by random permutation of sequences that shared a sampling date

**Fig. 3:**
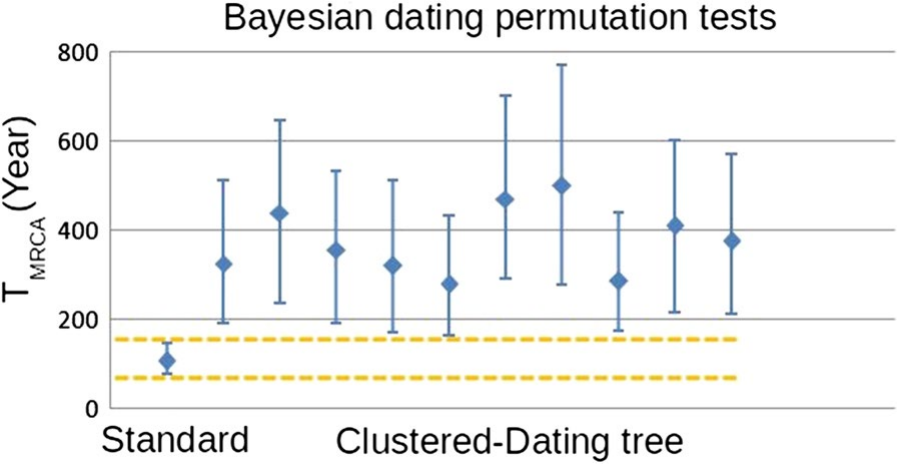
The maximum a posteriori estimate of the tMRCA with 95% highest posterior density intervals as inferred using BEAST. These are compared to equivalent estimates from data sets with the sampling sequences randomly permuted (clusters of sequences).The Y-axis indicates the tMRCA and the X-axis shows randomization of the data set, with the non-randomized data set (i.e., with dates correctly assigned to sequences) shown between horizontal yellow lines. The circles represent the mean tMRCA and the error bars show the 95% credible intervals

The likelihood mapping analysis showed that 85% of the dots fell at the corners of the triangles, whereas 11% of the dots fell in the central area, indicating that the alignment (the dataset) contained sufficient genetic information for the phylogenetic analysis (Fig. 1). Tip-dated analyses using the WNV-2 data set showed evidence of temporal structure, with R^2^ values of 0.76 (Fig. 2). Using clustered permutations, the 95% higher posterior density interval (HPD) of the rate did not overlap between the true and randomized data. Hence, the temporal signal in the WNV-2 data is sufficient to reliably estimate the substitution rate of the given data (Fig. 3).

### Estimated rates of WNV-2 complete genome evolution

The dated tree and the evolutionary rate of 75 complete genome sequences of WNV-2 were estimated using the MCMC approach, the GTR + G + I substitution model, and the uncorrelated lognormal relaxed clock assumption (2 lnBF = 9.75). Under the relaxed clock assumption, the BF analysis showed that the BSP fits better with the given data (2 lnBF > 40). The estimated mean value of the WNV-2 evolutionary rate was 3.46 × 10^−4^ substitution/site/year (95% HPD: 2.5 × 10^−4^–4.47 × 10^−4^).

We repeated our analyses excluding the sample of Senegal as a possible outlier, with nearly identical results (Additional file 1: Figure S2).

### Phylogenetics of WNV-2 complete genome

The ML tree identified two highly significant clades (A and B). Clade A represents the majority of the European isolates (Additional file 1: Figure S3), whereas clade B includes African isolates and one isolate from Ukraine. Two isolates, collected from South Africa (2008) and Zambia (2016), represent an outgroup outside the two main clades.

### Phylogeographic analysis of the full-length WNV-2 genome to trace virus spread

**Fig. 4:**
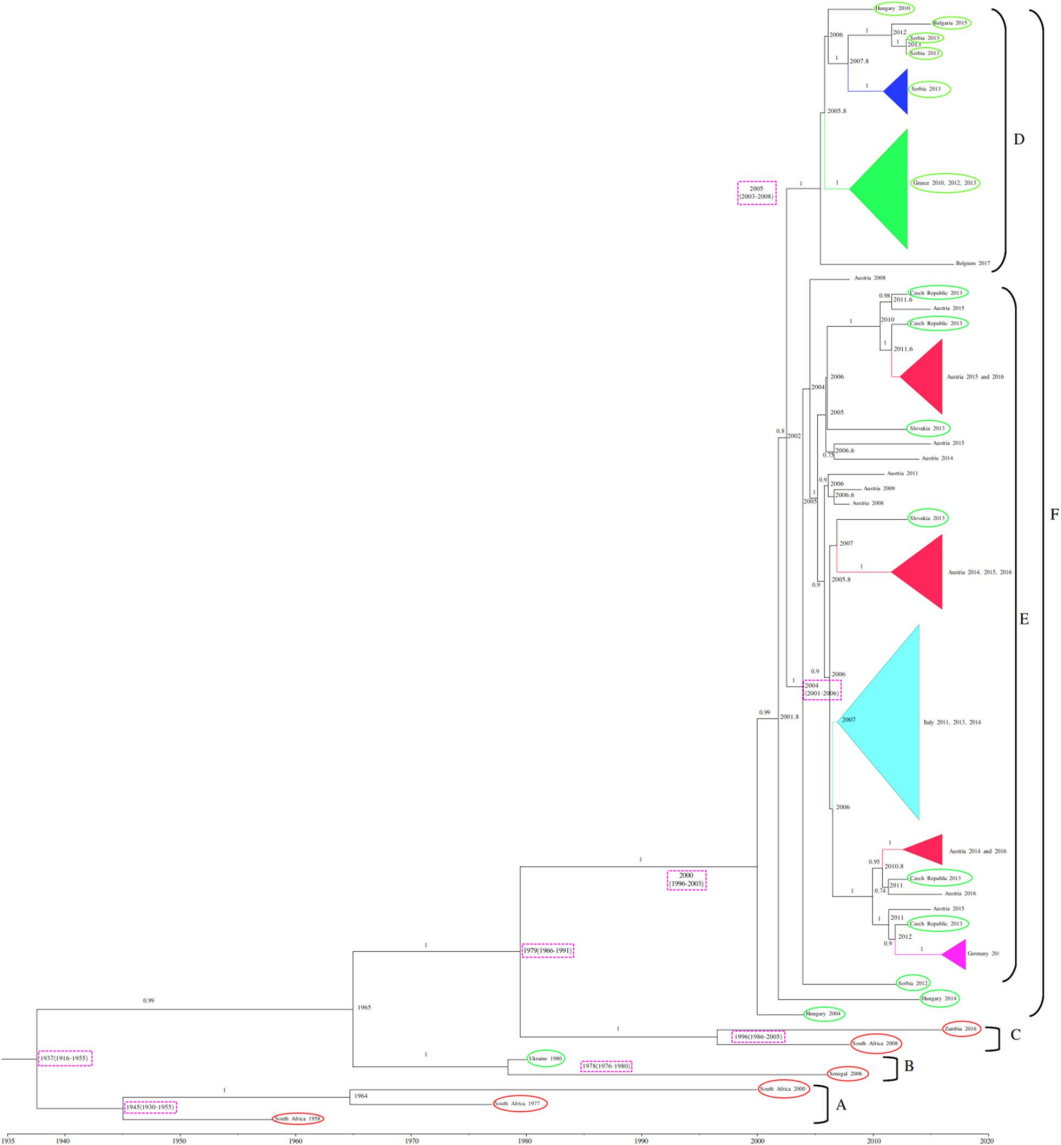
Bayesian phylogeographic tree of West Nile lineage 2 complete genome sequences. Main clades described by alphabets: clade A–F. Groups of sequences belonging to the same region were collapsed and colored. Strains from Africa were shown by red circles whereas strains from Europe were shown by green circles. Branch support was shown for posterior probability greater than 0.7. Years (95% HPD) are reported in the main nodes

**Fig. 5:**
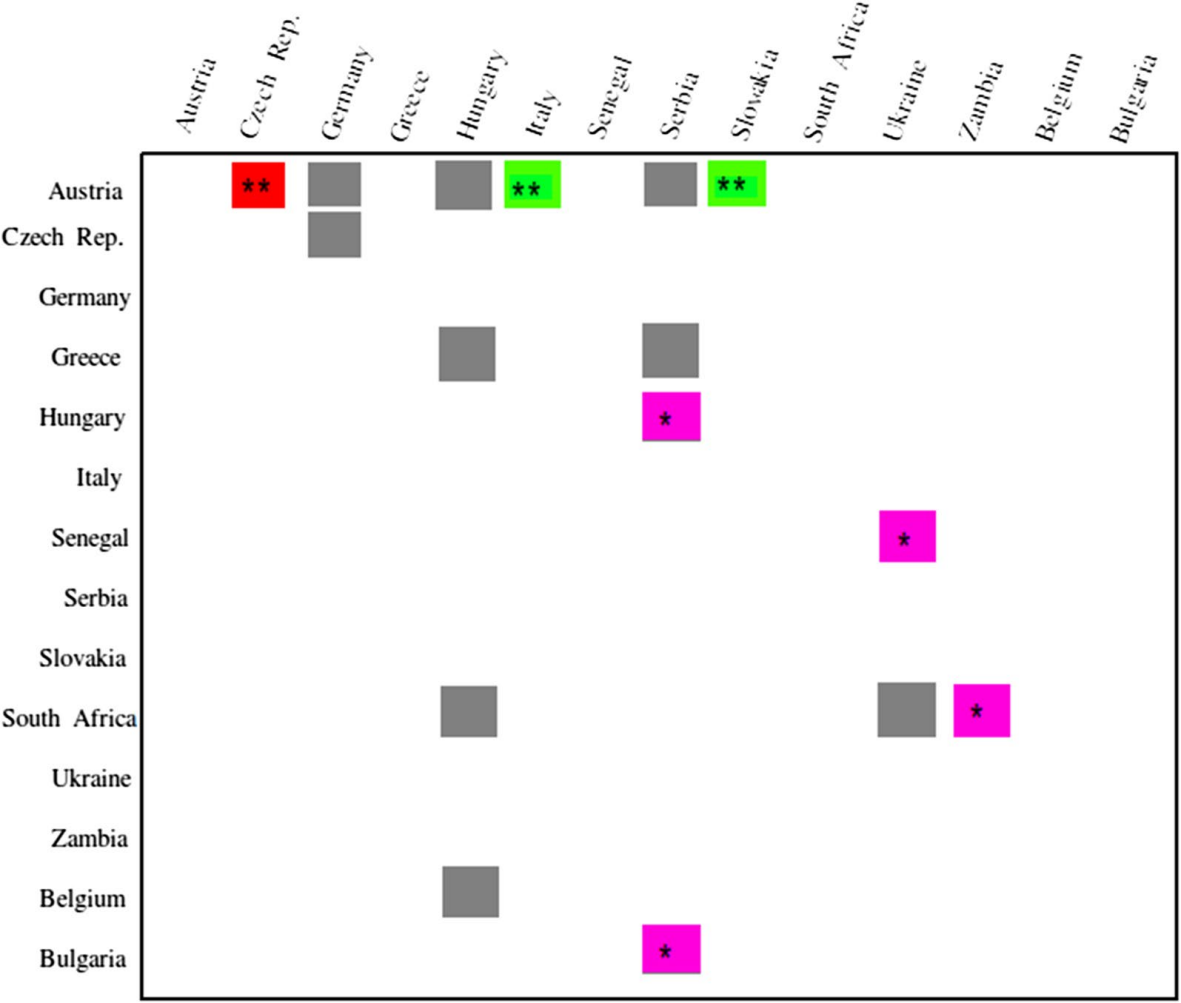
Level of Bayes Factor support for each transmission route. The levels of Bayes Factor (BF) support greater than 3 were shown by different colors: BF > 1000 (red), 100 < BF > 1000 (green), 10 < BF > 100 (magenta), and 3 < BF > 10 (grey). The Y-axis represents the origin location and the X-axis represents the destination. 0.7 < Posterior probability (PP) < 1.0 was shown by a star whereas PP = 1.0 was shown by two stars

**Fig. 6:**
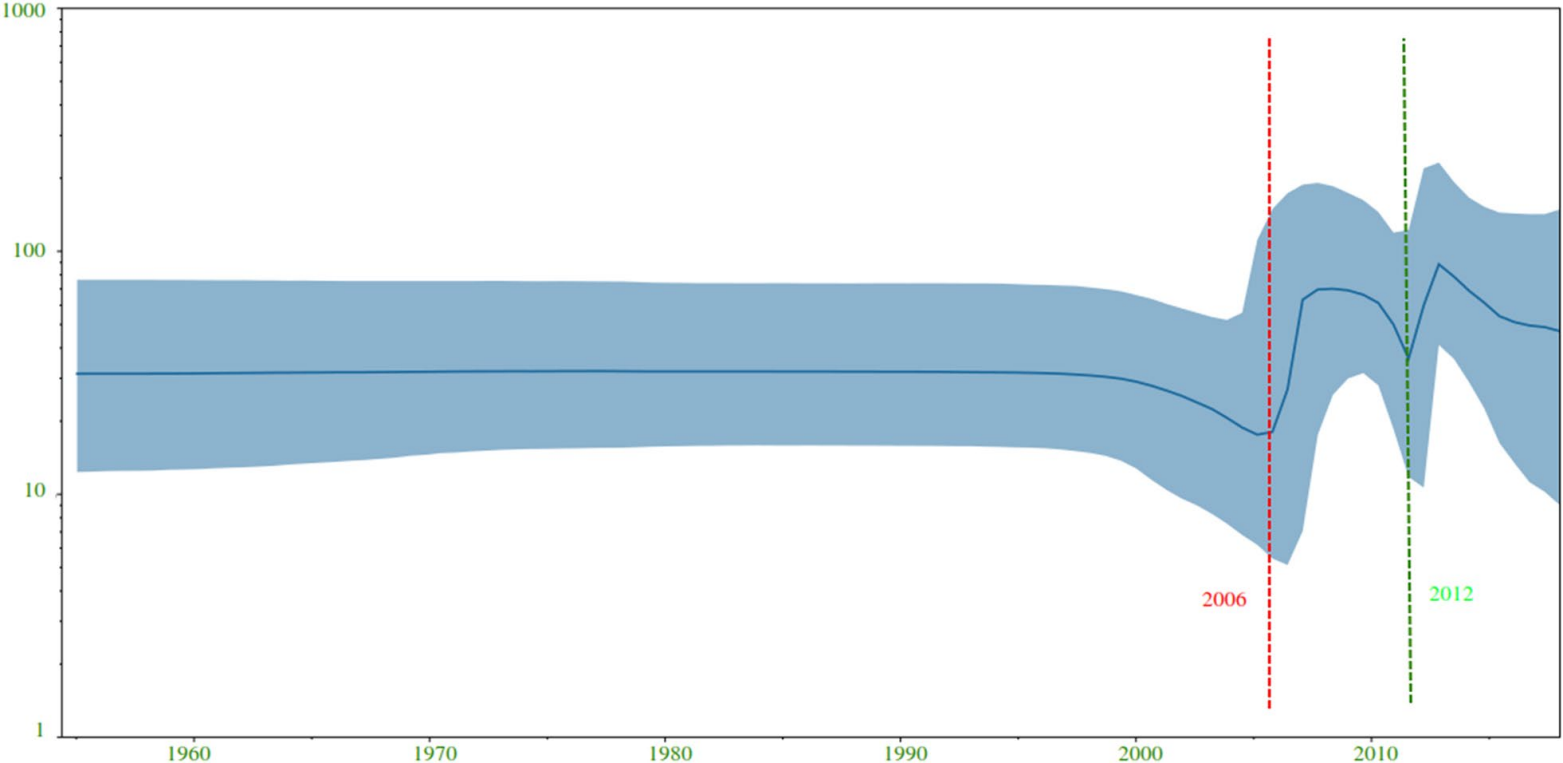
Population dynamics analysis of the WNV-2 isolates shown as Bayesian skyline plot (BSP). The effective number of infections is indicated on the Y axis, and time on the X-axis. The blue colored area corresponds to the credibility interval based on the 95% highest posterior density interval (HPD)

**Fig. 7:**
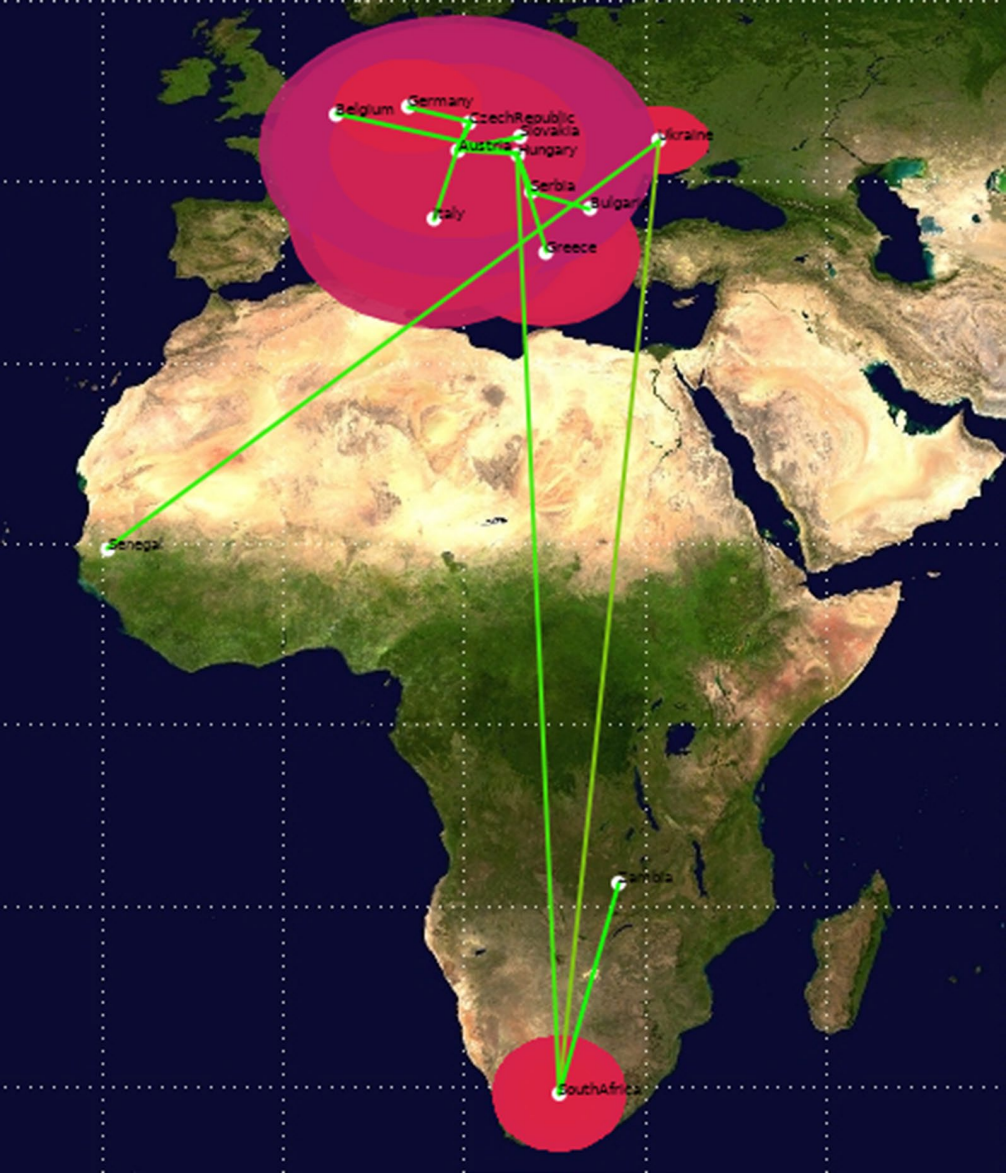
The lines connecting different locations represent branches in the MCC tree on which state exchanges occur and circle areas reflect the number of branches maintaining a particular state at that time point

The time to most recent common ancestor (tMRCA) estimates for WNV-2 were expressed as median and 95% HPD years before the most recent sampling date, corresponding to 2018 in this study. The Bayesian maximum clade credibility tree showed that the tMRCA of 83 years, corresponding to 1937 (95% HPD: 1916–1955). Four clades have been identified (A, B, C, and F) (Fig. 4). Clade A, dating back to 1945 (95% HPD: 1930–1955), included three isolates from South Africa; clade B, dated back to 1978 (95% HPD: 1976–1980), included two isolates from Africa (Senegal) and eastern Europe (Ukraine); clade C, dated back to 1996 (95% HPD: 1986–2005), included two isolates from Africa (South Africa and Zambia); clade F, dating back to 2000 (95% HPD: 1996–2003), included several isolates from Europe. Clade F contains further two subclades, D and E. Clade D, dating back to 2005 (95% HPD: 2003–2008), included isolates from eastern Europe (Serbia, Greece, Hungary, and Bulgaria) and a single isolate from western Europe (Belgium). Clade E, dating back to 2004 (95% HPD: 2001–2006), included isolates from several central/eastern European countries. We quantified patterns of WNV-2 spatial diffusion under the BSSVS procedure. While the most probable ancestral location of clade A was South Africa, the most probable ancestral location of clade B was either Senegal or Ukraine (state posterior probability [stpp] = 1.0). Similarly, the most probable ancestral location of clade C was either South Africa or Zambia (stpp = 1.0). The most probable ancestral location of clades F and D was Hungary (stpp = 1.0), whereas the most probable ancestral location of clade E was Serbia and/or Austria (equal stpp = 1.0). The levels of support for transitions using the BF cut-off value (BF > 3) were shown (Fig. 5). The results showed a highly supported transition from Austria to the Czech Republic (BF > 10,000); very strongly supported transitions from Austria to Italy and Slovakia (BF range from 100 to 200); strongly supported transitions from Greece to Hungary (BF = 11), from Senegal to Ukraine (BF = 27), from South Africa to Zambia (BF = 13), from Bulgaria to Serbia (BF = 91), and from Hungary to Serbia (BF = 80). Nonetheless, there was significant support for transitions from Austria to Germany (BF = 4), Hungary (BF = 9), and Serbia (BF = 3.6); the Czech Republic to Germany (BF = 7); Greece to Serbia (BF = 3.17); South Africa to Ukraine (BF = 8.8) and Hungary (BF = 3); and from Belgium to Hungary (BF = 7). The transmission routes of the WNV-2 were mapped and visualized using the SPREAD3 software (Additional file 1: Figure S4). The Skyline plot showed that the infected population size remained relatively constant in size until 2002, and then there was a partial decrease (Fig. 6). The results showed an exponential growth in two episodes (2006 and 2012). The data collected revealed two possible routes for the introduction of WNV-2 into Europe (Fig. 7); both pathways suggested South Africa or nearby regions as possible sources of introduction. One pathway showed that the isolates from South Africa spread northward to reach Hungary, then was redirected into neighboring countries (Additional file 2: Video S1). The second pathway showed that the Southern African isolates first reached Ukraine and then redirected south-westward to reach Senegal (Additional file 3: Video S2), probably through western Europe and North Africa. In summary, our results suggest a plausible scenario of the virus spreading from Africa to Europe. To identify a possible association between the results obtained in the present study and the available migratory bird data and to estimate the risk of transmission of the infection between Europe and Africa, we combined the distribution of the reported infected bird species with the results obtained in the present study. More than 2 billion birds travel between Europe and Africa, 73% of which are accounted for by just 16 species [26]. In the present study, we retrieved the available data for 14 migratory birds, the distribution-range of which extends from Europe to Africa. These are Barred Warbler (*Sylvia nisoria*)*,* European Turtle Dove (*Streptopelia turtur)* [18, 73, 77], Great Reed-warbler (*Acrocephalus arundinaceus)* [37, 40, 74], Sedge Warbler (*Acrocephalus schoenobaenus),* Common Kingfisher (*Alcedo atthis)* [31], Common Swift (*Apus apus)* [49, 81], European Pied Flycatcher (*Ficedula hypoleuca*) [41], Woodchat Shrike (*Lanius senator*), Eurasian Blackcap (*Sylvia atricapilla*) [33], White stork (*Ciconia ciconia)*, Black kite (*Milvus migrans)* [41, 49, 65], Common Nightingale (Luscinia megarhynchos), Willow Warbler (*Phylloscopus trochilus*) [33], and Lesser Spotted Eagle (*Aquila pomarina*) [65] (Additional file 1: Figure S5).

## Discussion

The present data set on a sample of 75 published complete genomes of WNV allowed for a valuable re-examination of the phylogeographic progress of the virus in recent years. The collected data showed an increase in the total number of WNV infections in the Mediterranean region and raises the question of the possible mode of transmission as well as the geographic distribution of the detected WNV-2 strains. A major advantage of phylogenetic analysis is the ability to infer the migration process at natural time scales. The present study showed that the mean evolutionary rate for the WNV-2 strains used was estimated at 3.46 × 10^−4^ substitution/site/year. Similar results were reported in studies involving strains from different continents (2.7 × 10^–4^ substitution/site/year) [48] and European strains (3.7 × 10^–4^ substitution/site/year) [12]. Zehender et al. [79] reported a slightly higher mean evolutionary rate (5.2 × 10^–4^ substitution/site/year). This difference could be attributed to the different datasets analyzed and the applied nucleotide substitutions and coalescent models.

The tMRCA estimates suggest that the European WNV-2 entered Europe as a result of at least two introduction events in 1978 (1976–1980; Ukraine) and in 2000 (1996–2003; clade F). The latter introduction date of WNV-2 was in line with the available epidemiological data reported previously [14, 79]. However, our results showed another introduction date, as early as 1978, from Africa (Senegal) to Ukraine or nearby regions. The first evidence of WNV-2 in Cyprus stems from a serological survey in the Eastern Mediterranean between 1966 and 1971 [77], before the detection date of WNV-2 in Hungary and close to the introduction date in our study.

WNV-2 strains detected in Ukraine are not well correlated to the remaining European countries, whereas the strains detected in Hungary constitute an ecological niche and have a central role in the dissemination of strains throughout Europe.

If we limit the WNV-2 transition to countries showing the stpp greater than 70%, the analysis of the phylogeographical tree showed that the WNV-2 migration was from Austria to the Czech Republic, Italy and Slovakia. These findings are supported by a phylogenetic analysis of WNV-2 strains collected from the Czech Republic, demonstrating that the Czech WNV-2 strains isolated during August 2013 were closely related to Austrian, Italian and Serbian strains reported in 2008, 2011 and 2012, respectively [63]. Furthermore, a closer phylogenetic relationship was observed between the Italian WNV-2 strains identified in 2013–2014 and those from Austria and the Czech Republic [6]. In Slovakia, partial NS5 gene analysis showed almost 100% identity of WNV-2 strains detected in Komárno district with strains from Germany, Italy, and Austria [11].

Moreover, WNV-2 migration was shown from Hungary and Bulgaria to Serbia. A previous study revealed that the re-emergence of WNV-2 in Northern Greece in 2018 involved its spread from Hungary through Serbia and, then, through Bulgaria [12].

Furthermore, the analysis showed WNV-2 migration from South Africa to Zambia. Orba et al. [53] reported the first WNV-2 case in Zambia, which was closely related genetically to the WNV-2 South African strains. The last significant WNV-2 migration pathway was from Senegal to Ukraine. A recent phylogeography and spatio-temporal dispersal pattern analysis of WNV-2 in the Danube Delta Biosphere Reserve, which small parts are also located in the Ukraine, showed that Romania has experienced a WNV-2 introduction event from South Africa or Senegal [72].

Altogether, our phylogeographical analysis of the migration of WNV-2 indicated Hungary and Austria as radiation centres of European WNV-2, whereas the remaining European countries mainly acted as receiving areas. The present study showed relationships between WNV-2 strains from Europe and Africa.

In South Africa, as an endemic area, wild birds are considered the main reservoirs for WNV-2 and other related Flaviviruses [25, 34, 46, 47]. The South African prototype WNV-2 isolate (H 442) used in the present study was detected in 1958 in the blood of a person with a mild febrile disease who had been bitten by mosquitoes while catching birds in mist nets for arbovirus studies [36]. The first most correlated isolate to the South African one, used in the present study, was the Ukrainian strain (LEIV-3266Ukr); the latter strain was isolated from the blood and internal organs of a wild bird (Rook, *Corvus frugilegus*) in 1980, in the territory of the Black Sea State Preserve of the Ukrainian Academy of Sciences (the Kherson region) [75]. The infection could have been introduced to Europe by infected migratory birds as they are considered to act as carry-over vectors and may be one mode of introduction for viruses into new regions and countries [8]. However, the role of migratory birds in the transmission of WNV-2 is the subject of continuing debate and has received considerable attention following the epidemics in Europe and North America [59].

The role of migratory birds in the introduction of the virus (lineage 1 or lineage 2) into Europe and the Mediterranean Basin is clearly assessed by numerous studies in Spain [19, 21–23, 32, 42], Palestine [43], Poland [31], Romania [52], and the Czech Republic [31]. However, long-distance migratory birds are not necessary to explain the spread of WNV-2 over large distances; the virus could have been moved to shorter distances sequentially by multiple individuals of short- and long-distance migratory infected species. In the USA, [15] developed a mathematical model for the transmission of WN. They found that yearly seasonal outbreaks depend primarily on the number of susceptible migrant birds entering the local population each season, yet reported that the early growth rates of seasonal outbreaks are more influenced by the migratory population than by the resident bird population.

The role of migratory birds in WNV-2 infection needs to be further studied by more advanced techniques and through joint projects between European and African researchers. Birds’ migration routes are not random. When traveling between their breeding and wintering grounds, migratory birds follow set routes that include suitable habitats. The African-European flyway connects Europe's breeding grounds with Africa’s wintering grounds, including vital stop-over sites in the Middle East and the Mediterranean.

Interestingly, far from the Mediterranean region, a recent study in Malaysia reported that the WNV-2 strains detected in local wild birds have a 99% similarity to the strains from South Africa [2], enhancing the need to consider migratory birds in future epidemiological analysis tracking WNV-2 infections.

Phylogeography with far greater numbers of samples will lead to detailed insights about paths of transmission across species and geographic regions.

## Conclusion

In summary, our findings presented here indicate that WNV-2 strains used in our case and found in Europe are phylogenetically related to African strains. The WNV-2 might have been introduced into Europe earlier than reported previously through multiple waves. Although these strains are most probably dispersed by migrating birds via the Mediterranean flyway, international surveillance cooperation would be required to follow in real time the dispersion of emergent WNV-2 strains having the potential of causing increased mortality and morbidity in human and wildlife populations. Further and more specific studies would also be useful to fill in some existing gaps in our current knowledge of WNV-2 epidemiology and to improve disease control.

## Supplementary Information

**Additional file 1. Table S1:** List of sequences used in our study. **Figure S1:** Available data of WNV-2 sequences collected from 2008 until October 2018. **Figure S2:** Obtained results without the Senegal sample (as a possible outlier). **Figure S3:** The two highly significant clades (A and B) identified with Maximum-likelihood tree. **Figure S4:** Transmission routes of WNV-2 mapped and visualized using SPREAD3. **Figure S5:** Combination of the distribution of the reported infected bird species with the results obtained in the present study.

**Additional file 2. Video S1:** First pathway from South Africa to Hungary and neighboring countries.

**Additional file 3. Video S2:** Second pathway from South Africa to Ukraine and Senegal.

## Data Availability

Data were collected from NCBI and filtered carefully. If someone want to request the data used in this study please send an email to: srihi.haythem@gmail.com/nabil.abid@isbst.uma.tn. The GenBank accession numbers for the sequences reported in this paper are: JN393308, JN858070, JX041631, KC407673, KC496015, HQ537483, KF179639, KF179640, KF588365, KF647249, DQ116961, DQ318019, EF429199, EF429200, KF647251, KF823806, KJ883343, KJ883344, KJ883345, KJ883346, KJ883348, KJ883349, KM052152, KM203860, KM203861, KM203862, KM203863, KM659876, KP109691, KP109692, KP780837, KP780838, KP780839, KP780840, KP789953, KP789954, KP789955, KP789956, KP789957, KP789958, KP789959, KP789960, KT207792, KT359349, KT757318, KT757319, KT757320, KT757321, KT757322, KT757323, KU206781, KY594040, LC318700, MF984337, MF984338, MF984339, MF984340, MF984341, MF984342, MF984343, MF984344, MF984345, MF984346, MF984347, MF984348, MF984349, MF984350, MF984351, MF984352, MH021189, MH244512, MH244513, MH924836, MH986055, MH986056.

## Abbreviations

2 lnBF: 2 × Log Bayes factors
BEAST: Bayesian evolutionary analysis sampling trees
BEAUTi: Bayesian evolutionary analysis utility
BF: Bayes factors
BIC: Bayesian information criterion
BSSVS: Bayesian stochastic search variable selection
CTMC: Continuous-time Markov chain
ESS: Effective sample size
G: Gamma
GTR: General time reversible
H0: Null hypothesis
HPD: Higher posterior density interval
I: Invariable sites
IRD: Institute of Research for Development
IUCN: International Union for Conservation of Nature
KML: Keyhole markup language
LBP: Local bootstrap probability
MCC: Maximum clade credibility
MCMC: Bayesian Markov Chain Monte Carlo
ML: Maximum-likelihood
MLE: Marginal likelihood estimators
NNI: Nearest neighbor interchange
PAUP: Phylogenetic analysis using the PAUP
PS: Path sampling
RDP4: Recombination detection program 4
SH-like aLERT: Shimodaira–Hasegawa-like approximate likelihood ratio test
SPR: Subtree pruning and regrafting
SPREAD3: Spatial phylogenetics reconstruction of evolutionary dynamics using data-driven documents (D3)
SS: Stepping-stones sampling
tMRCA: Time to most recent common ancestor
UFBoot: Ultrafast bootstrap
WNV: West Nile virus
