# Supplementary material for: Phylodynamic and phylogeographic analysis of the complete genome of the West Nile virus lineage 2 (WNV-2) in the Mediterranean basin
Source: BMC Ecol Evol. 2021 Sep 27;21:183. doi: 10.1186/s12862-021-01902-w (PMC8477494; doi:10.1186/s12862-021-01902-w)
Supplement: Supplementary file 1 — Additional file 1. Table S1: List of sequences used in our study. Figure S1: Available data of WNV-2 sequences collected from 2008 until October 2018. Figure S2: Obtained results without the Senegal sample (as a possible outlier). Figure S3: The two highly significant clades (A and B) identified with Maximum-likelihood tree. Figure S4: Transmission routes of WNV-2 mapped and visualized using SPREAD3. Figure S5: Combination of the distribution of the reported infected bird species with the results obtained in the present study. [file 12862_2021_1902_MOESM1_ESM.docx]

**List of supplementary figures/table**


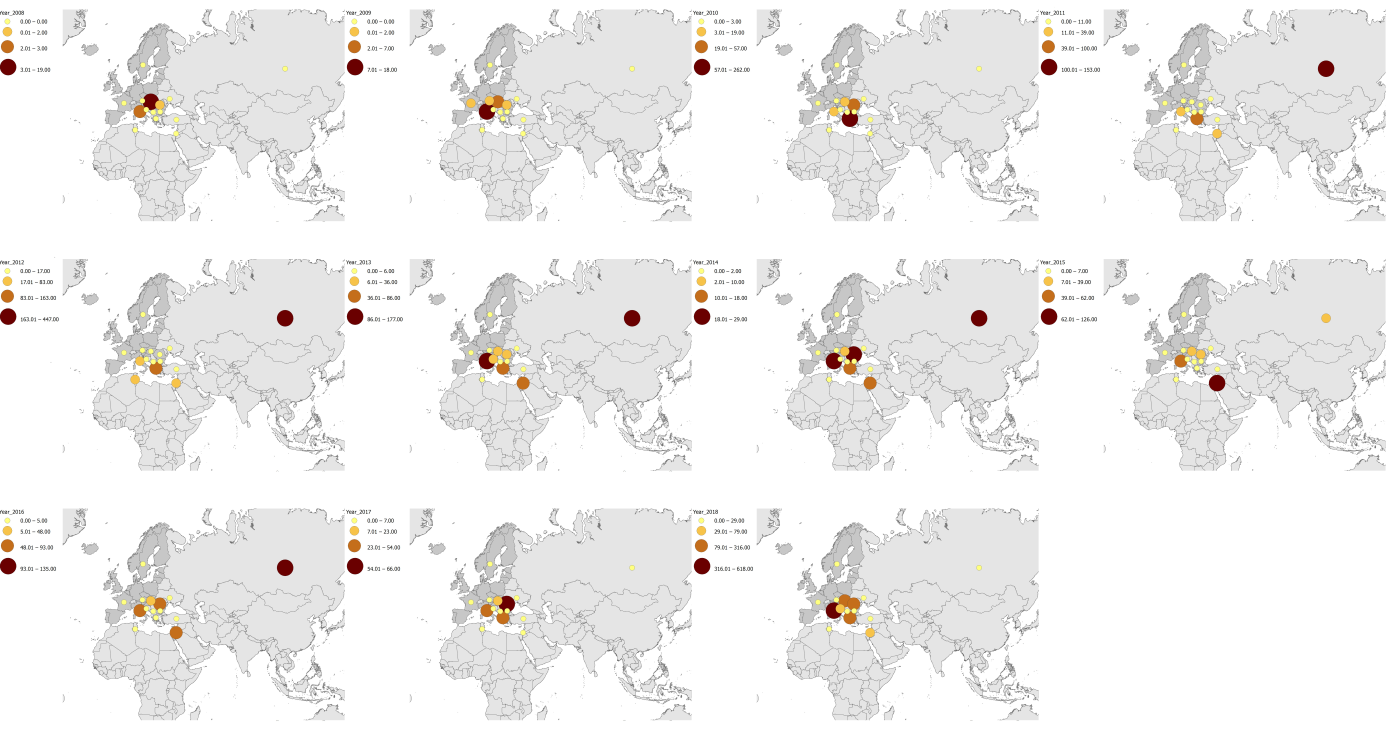

**Figure S1.** Available data of WNV-2 sequences collected from 2008 until October 2018.


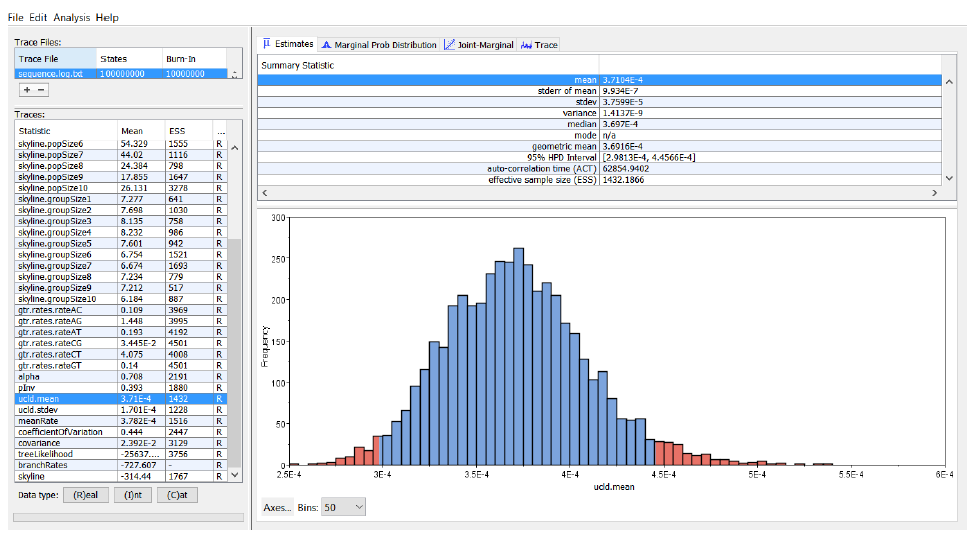


**Figure S2.** Obtained results when we repeated our analyses excluding the sample of Senegal as a possible outlier.


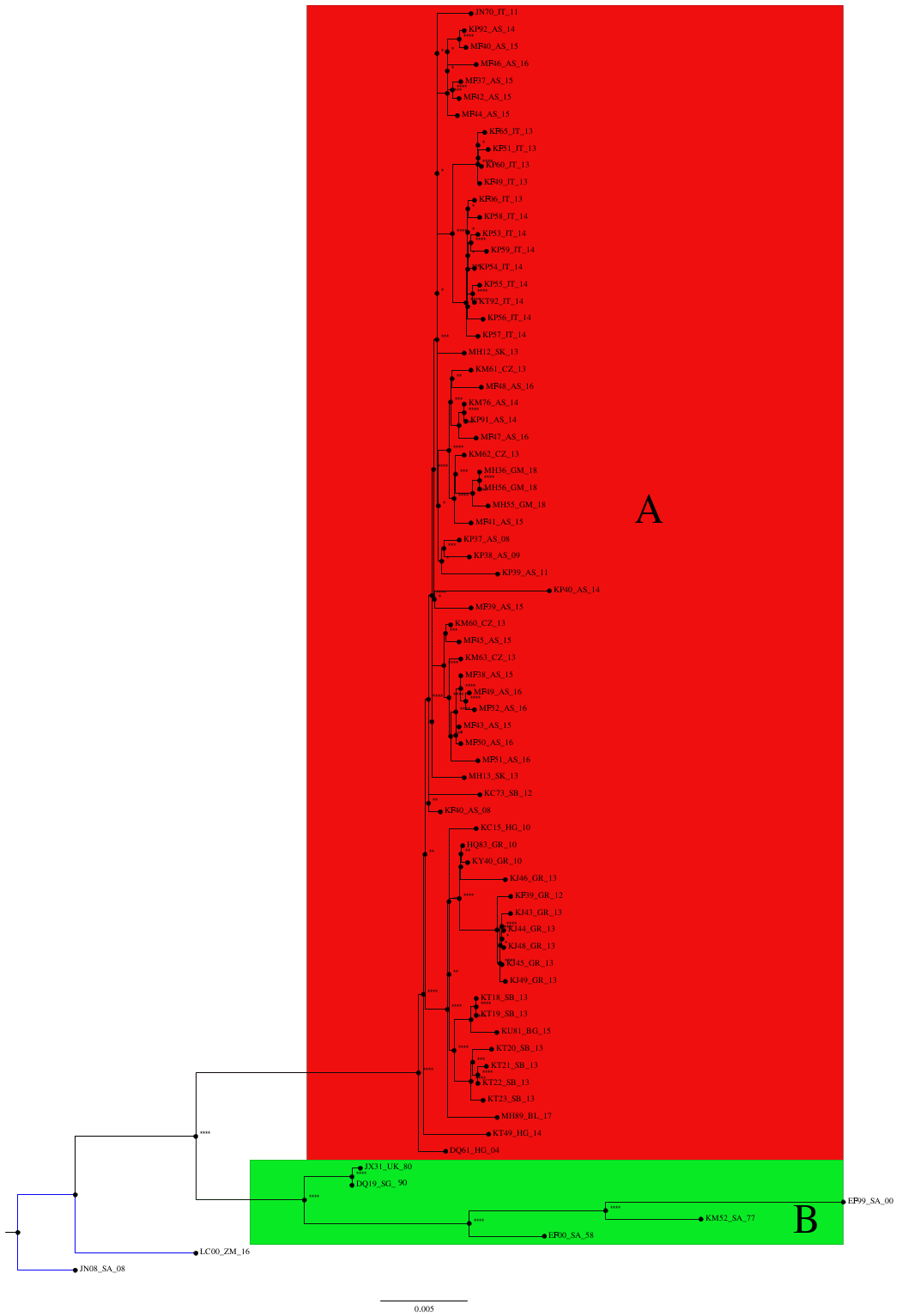


**Figure S3.** The two highly significant clades (A and B) identified with Maximum-likelihood tree. Clade A represents the majority of the European isolates, whereas clade B includes African isolates and one isolate from Ukraine. Two isolates, collected from South Africa (2008) and Zambia (2016), represent an outgroup outside the two main clades.


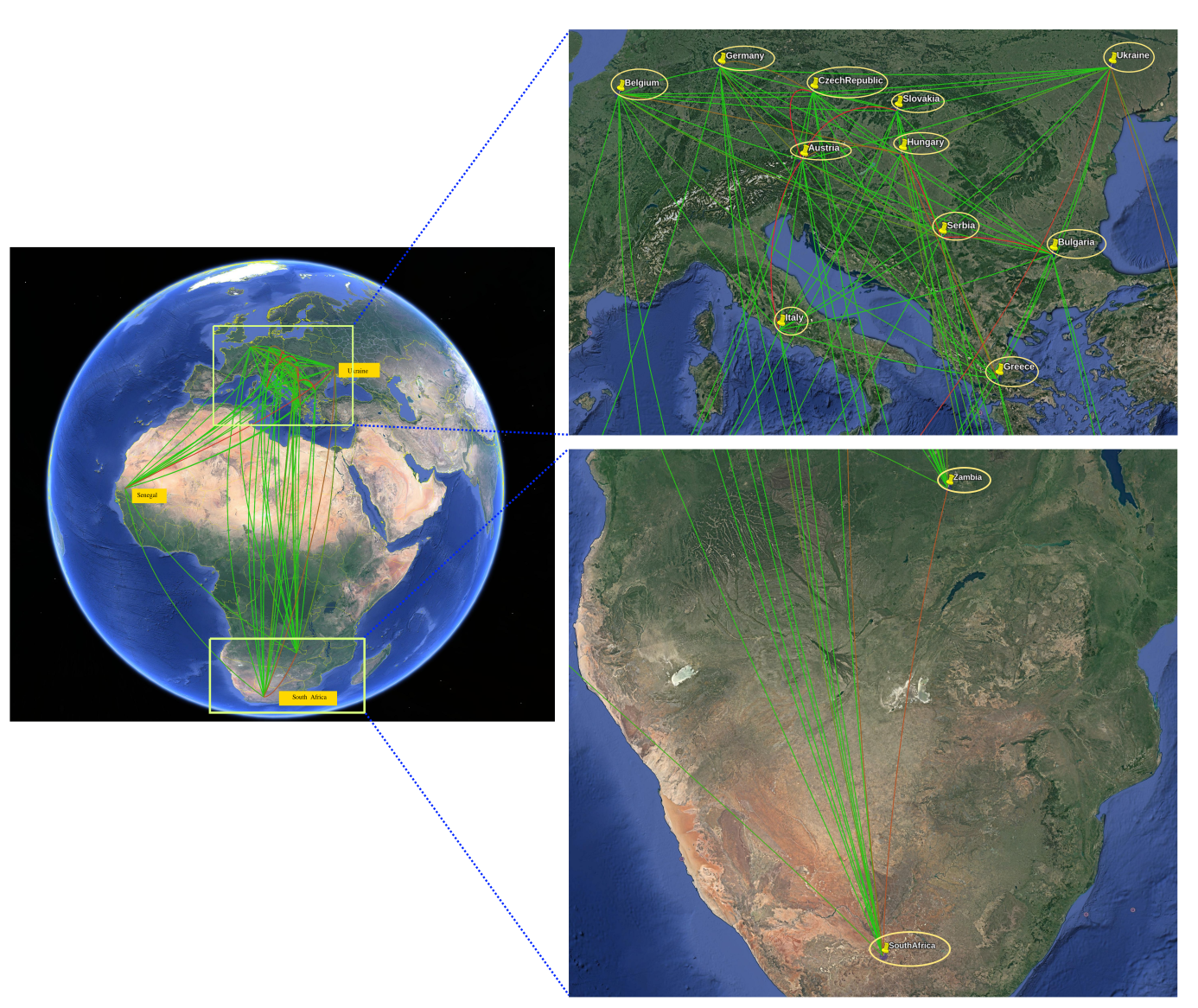


**Figure S4.** The transmission routes of the WNV-2 mapped and visualized using the SPREAD3 software.


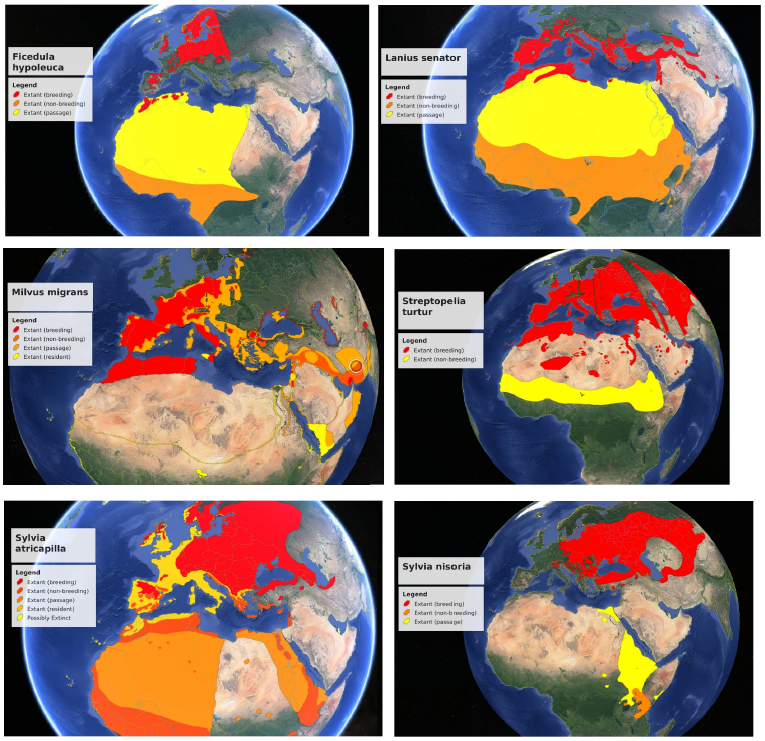


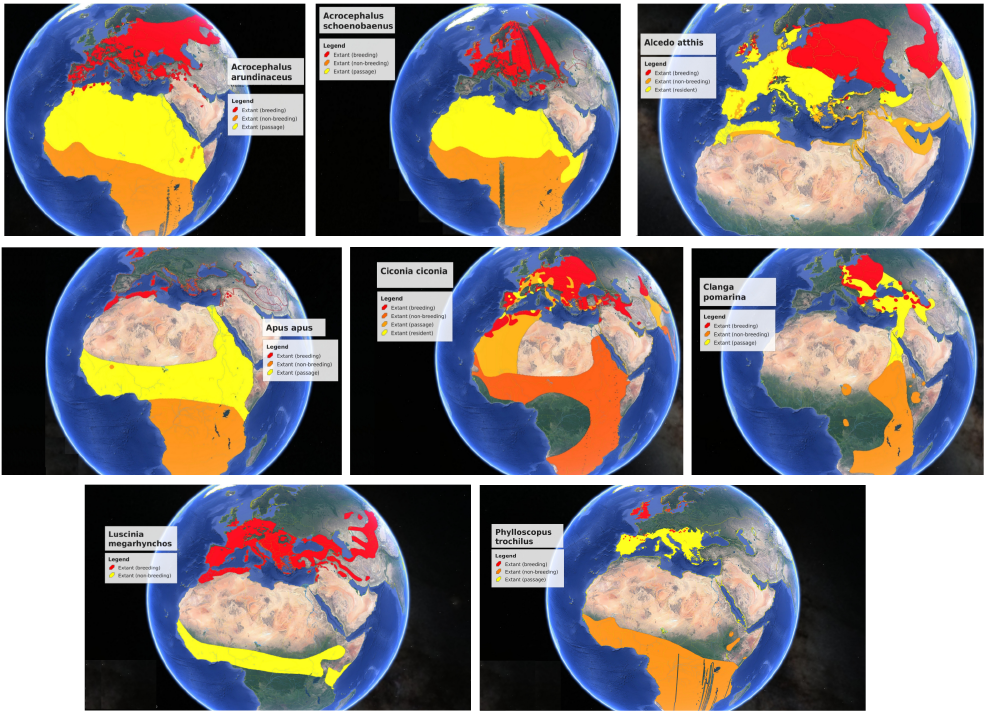


**Figure S5.** Combination of the distribution of the reported infected bird species with the results obtained in the present study.

**Table S1**

| **Nucleotide sequence accession numbers** | **Country** | **Collection date** | **Host** |
| --- | --- | --- | --- |
| JN393308 | SouthAfrica | 2008 | Horse |
| JN858070 | Italy | 2011 | Homo sapiens |
| JX041631 | Ukraine | 1980 | Bird |
| KC407673 | Serbia | 2012 | Northern goshawk |
| KC496015 | Hungary | 2010 | Horse |
| HQ537483 | Greece | 2010 | Culex pipiens |
| KF179639 | Greece | 2012 | Homo sapiens |
| KF179640 | Austria | 2008 | goshawk |
| KF588365 | Italy | 2013 | Homo sapiens |
| KF647249 | Italy | 2013 | Homo sapiens |
| DQ116961 | Hungary | 2004 | Goshawk |
| DQ318019 | Senegal | 2006 | Mosquito: (Mimomyia lacustris) |
| EF429199 | SouthAfrica | 2000 | Homo sapiens |
| EF429200 | SouthAfrica | 1958 | Homo sapiens |
| KF647251 | Italy | 2013 | Homo sapiens |
| KF823806 | Italy | 2013 | Homo sapiens |
| KJ883343 | Greece | 2013 | Homo sapiens |
| KJ883344 | Greece | 2013 | Homo sapiens |
| KJ883345 | Greece | 2013 | Homo sapiens |
| KJ883346 | Greece | 2013 | Homo sapiens |
| KJ883348 | Greece | 2013 | Homo sapiens |
| KJ883349 | Greece | 2013 | Homo sapiens |
| KM052152 | SouthAfrica | 1977 | Horse |
| KM203860 | CzechRepublic | 2013 | Culex modestus |
| KM203861 | CzechRepublic | 2013 | Culex modestus |
| KM203862 | CzechRepublic | 2013 | Culex modestus |
| KM203863 | CzechRepublic | 2013 | Culex modestus |
| KM659876 | Austria | 2014 | Homo sapiens |
| KP109691 | Austria | 2014 | Homo sapiens |
| KP109692 | Austria | 2014 | Culex pipiens |
| KP780837 | Austria | 2008 | Nestor notabilis (kea) |
| KP780838 | Austria | 2009 | Nestor notabilis (kea) |
| KP780839 | Austria | 2011 | Nestor notabilis (kea) |
| KP780840 | Austria | 2014 | Nestor notabilis (kea) |
| KP789953 | Italy | 2014 | Homo sapiens |
| KP789954 | Italy | 2014 | Homo sapiens |
| KP789955 | Italy | 2014 | Homo sapiens |
| KP789956 | Italy | 2014 | Homo sapiens |
| KP789957 | Italy | 2014 | Homo sapiens |
| KP789958 | Italy | 2014 | Homo sapiens |
| KP789959 | Italy | 2014 | Homo sapiens |
| KP789960 | Italy | 2013 | Homo sapiens |
| KT207792 | Italy | 2014 | Mosquito |
| KT359349 | Hungary | 2014 | Homo sapiens |
| KT757318 | Serbia | 2013 | Culex pipiens |
| KT757319 | Serbia | 2013 | Culex pipiens |
| KT757320 | Serbia | 2013 | Culex pipiens |
| KT757321 | Serbia | 2013 | Culex pipiens |
| KT757322 | Serbia | 2013 | Culex pipiens |
| KT757323 | Serbia | 2013 | Culex pipiens |
| KU206781 | Bulgaria | 2015 | Homo sapiens |
| KY594040 | Greece | 2010 | Homo sapiens |
| LC318700 | Zambia | 2016 | Culex quinquefasciatus |
| MF984337 | Austria | 2015 | Homo sapiens |
| MF984338 | Austria | 2015 | Homo sapiens |
| MF984339 | Austria | 2015 | Homo sapiens |
| MF984340 | Austria | 2015 | Homo sapiens |
| MF984341 | Austria | 2015 | Homo sapiens |
| MF984342 | Austria | 2015 | Homo sapiens |
| MF984343 | Austria | 2015 | Homo sapiens |
| MF984344 | Austria | 2015 | Goshawk |
| MF984345 | Austria | 2015 | Falcon |
| MF984346 | Austria | 2016 | Homo sapiens |
| MF984347 | Austria | 2016 | Homo sapiens |
| MF984348 | Austria | 2016 | Homo sapiens |
| MF984349 | Austria | 2016 | Horse |
| MF984350 | Austria | 2016 | Horse |
| MF984351 | Austria | 2016 | Culex pipiens |
| MF984352 | Austria | 2016 | Culex pipiens |
| MH021189 | Belgium | 2017 | Homo sapiens |
| MH244512 | Slovakia | 2013 | Northern goshawk |
| MH244513 | Slovakia | 2013 | Eurasian sparrow hawk |
| MH924836 | Germany | 2018 | Strix nebulosa |
| MH986055 | Germany | 2018 | Turdus merula |
| MH986056 | Germany | 2018 | Turdus merula |
